# A web-based survey of contact lens-related adverse events among the Japanese female population

**DOI:** 10.1038/s41598-021-95318-7

**Published:** 2021-08-05

**Authors:** Koichi Ono, Akira Murakami, Yuji Haishima

**Affiliations:** 1 Department of Ophthalmology, Juntendo University Graduate School of Medicine, 2-1-1 Hongo Bunkyo-ku, Tokyo, Japan 113-8421; 2 Department of Ophthalmology, Juntendo Tokyo Koto Geriatric Medical Center, Juntendo University School of Medicine, Tokyo, Japan; 3 Division of Medical Devices, National Institute of Health Science, Kawasaki, Japan

**Keywords:** Epidemiology, Eye diseases

## Abstract

To assess the safety of cosmetic contact lenses and to identify other factors of contact lens (CL)-related complications for Japanese females. A web-based, cross-sectional, observational survey of complications related to CL use was performed. The frequencies of complications were compared between transparent and cosmetic CLs. Besides lens pigmentation, age, replacement schedule, total experience, daily wear time, location of purchase, stacking of CLs, CL exchange with friends, compliance to hygiene procedure, replacement of CLs at intervals longer than recommended, and CL wear overnight were considered as risk factors. Logistic regression analyses were performed to calculate the odds ratios. A total of 3803 Japanese females were analyzed. The frequency of adverse events was 33.4% (95%CI 31.3–35.4%) and 35.7% (95%CI 33.5–38.0%) for transparent and cosmetic CLs, respectively. In a multivariate model, statistically significant factors associated with complications included the following: quarterly schedule lenses, replacement at intervals longer than recommended, compliance to hygiene procedure, overnight wearing, purchase at physical shops and on the internet, and longer daily wearing time. Most of the risk-increasing behaviors are preventable. The role of public health ophthalmology is to increase awareness and to improve CL use behaviors.

## Introduction

Cosmetic contact lenses (CCLs) appeared in the USA in the early 1980s and were previously prescribed to mask eye flaws and improve the cosmetic appearance of the eye(s)^1^. Today, CCLs are fast becoming an essential fashion item, especially for young females. CCL wearers make up a significant and growing proportion of the contact lens (CL) wearing population in Asian countries^2^.

The safety of cosmetic CLs is controversial. More specifically, there have been several negative reports about adverse events related to the use of CCLs^3–5^. However, a recent multisite prospective surveillance study of corneal infection in Asian countries indicated that CCL users with keratitis did not employ risk-aversion behaviors as compared to users of transparent CL^6^. Therefore, differences in the behavior pattern between CCL and transparent CL users, as well as pigmentation in a CL, might influence differences in CL-related eye disorders between the two groups.

A review of existing literature revealed that some aspects of CL use behaviors were strongly associated with adverse events related to CL use^2–8^. Regarding safety assessment in clinical trials, we surmise that during the clinical trial period, CL users would be uncharacteristically motivated to keep good hygiene behavior under the supervision of CL specialists. This behavior is in direct contrast to non-compliance reports that vary from 40 to 91% in real world settings^9–12^. The objectives of this study were to assess the safety of CCLs, and to investigate other independent risk factors of CL-related complications for Japanese females of reproductive age through online survey methods.

## Methods

Two sets of questionnaires were prepared by the survey team. The first was a screening test to recruit participants from internet users. The second was a detailed survey regarding the personal incidence of CL-related eye complications, features of the CL purchase location, and personal CL use behaviors.

The preliminary screening survey included: age, sex, type of CL in current use (CCL, transparent CL, or hard CL), the replacement schedule (daily, weekly, bi-weekly, monthly, quarterly, annually, or others/unknown), total experience with CLs (years), daily wear time (hours). The subsequent survey asked about the type of CL in current use (CCL, transparent CL, or both), the location of CL purchase (physical shop, internet, or eye clinic/hospital), their personal experience of CL related eye problems defined as the history of seeking eye care by acute tearing, eye/lid pain, and blurred vision in the previous year (yes or no), and their practice of the following behaviors: stacking CLs during wearing, exchanging CLs among friends, replacing CLs at longer intervals than recommended, sleeping while wearing CLs, and hygiene-related behaviors. To assess hygiene-related behavior, three questions were presented: (1) rinsing and/or preserving with tap water, (2) rinsing and/or preserving with expired disinfectant solution, (3) using the same CL case for more than three months. In our survey, we defined CCLs as CLs which change the appearance of the eyes (e.g., color enhancement CLs, opaque CLs, and limbal circle CLs). For individuals who only use daily disposable CL, the frequency (never, rarely, sometimes, or always) of re-use was assessed. Visibility tinted CLs, which include a small amount of dye, were defined as transparent CLs because they do not alter the natural color of your eyes.

Females who wore both CCLs and transparent CLs were considered within the CCL group. Individuals who answered “never” for the hygiene-related questions were categorized as “good” compliance, while those who answered other than “never” were categorized as “bad” compliance. A composite compliance to hygiene measure was created for each participant based on the number of answers categorized as “good” compliance in the three key behavior questions; the composite compliance scores ranged from zero (bad) to 3 (good). For individuals who wore only daily disposable CL, compliance was based on the frequency of re-use (never: 3, rarely: 2, sometimes: 1, always: 0).

The web-based survey was conducted in November 2018 through a contract with a Japanese professional marketing research *company *(Cross Marketing Ltd.) that was experienced in many academic research studies. We distributed an invitation link to selected female online panelists aged 16–49 with a similar distribution as the Japanese population. Once the invitation was received, invitees provided informed consent and voluntarily took part in the preliminary test. Individuals who wore soft CLs for therapeutic purposes or had a medication history of any chronic eye disease during the past year were excluded from the screening survey. Of the respondents eligible for all screening tests, 4000 individuals are randomly selected to proceed to the second stage of the survey.

### Statistical analyses

We used Student’s t-tests to assess differences among continuous data variables and χ^2^ tests for categorical variables comparisons between two groups. Age, total experience with CLs, and daily wear time were treated as continuous variables, while the other variables from the surveys were treated as categorical. Simple and multivariate logistic regression analyses were performed to calculate crude and adjusted odds ratios (ORs) and 95% confidence intervals (95%CIs) to assess the risk associated with adverse events related to CL use.

A *P *value < 0.05 in each statistical test for differences between populations was accepted as significant. STATA SE/15.1 for Windows (STATA CORP LLC, Texas, USA) was used for statistical analyses.

### Ethical considerations

The protocol of this study was approved by the Institutional Review Board of Juntendo University, School of Medicine, and the National Institute of Health Sciences. All participants provided informed consent electronically prior to commencing the survey. It was also obtained from the parents/legally authorized representatives when subjects were under 18. The study adhered to the tenets of the Helsinki Declaration.

## Results

**Figure 1:**
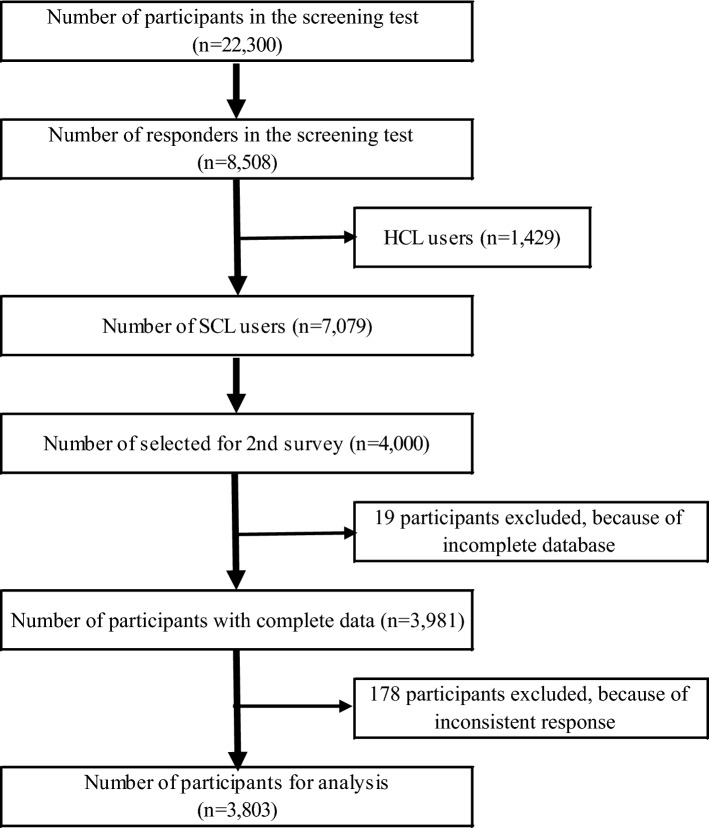
Flow diagram of study participants. HCL, hard contact lens; SCL, soft contact lens.

The screening test was nationally distributed to 22,300 randomly selected, panels of females aged 16–49. We received 8508 responses. We redistributed a detailed survey questionnaire to 4000 randomly selected individuals. Those with an incomplete survey (n = 19) and surveys with an inconsistent response between a screening test and second resurvey (n = 178) were excluded (Fig. 1). After exclusions, a total of 3803 surveys were analyzed in this study (response rate: 95.1%).

The mean age ± standard deviation of participants in this study was 28.7 ± 7.7 (Table 1). Tables 1 and 2 show the differences between the transparent and the cosmetic groups. Overall, 34.5% (95%CI 33.0–36.0%) of the females surveyed experienced CL-related eye problems in the previous year. The frequency of eye complication(s) was 33.4% (95%CI 31.3–35.4%), and 35.7% (95%CI 33.5–38.0%) for transparent and cosmetic group, respectively, and did not differ significantly between the CL types (p > 0.05).

**Table 1:** Characteristic of studied population by lens type (continuous variables).

	All (n = 3803)	Transparent CL (n = 1970)	Cosmetic CL (n = 1833)	*P* value (t test)
Age	28.7 ± 7.7	29.0 ± 8.0	28.4 ± 7.5	0.0084
Total experience (years)	11.1 ± 7.4	11.4 ± 7.8	10.8 ± 7.0	0.0062
Daily wear time (h)	11.2 ± 3.8	11.7 ± 3.6	10.7 ± 3.9	0.0000

Mean ± SD; CL, contact lens.

**Table 2:** Characteristic of studied population by lens type (categorical variables).

	ALL (n = 3803)		Transparent CL (n = 1970)		Cosmetic CL (n = 1833)		*P* value (χ^2^ test)
	n	%	n	%	n	%	
**Replacement schedule**							
Daily	1985	52.2	895	45.4	1090	59.5	0.003
Bi-weekly	1188	31.2	918	46.6	270	14.7	
Monthly	380	10.0	83	4.2	297	16.2	
Quarterly	36	1.0	5	0.3	31	1.7	
One year	179	4.7	46	2.3	133	7.3	
Others	35	0.9	23	1.2	12	0.7	
**Location of purchase**							
Eye clinic/hospital	1587	41.7	1058	53.7	529	28.9	0.000
Physical shop	786	20.7	414	21.0	372	20.3	
Internet	1430	37.6	498	25.3	932	50.9	
**Stacking of CLs**							
No	3609	94.9	1883	95.6	1726	94.2	0.047
Yes	194	5.1	87	4.4	107	5.8	
**CL exchange with friends**							
No	3647	95.9	1903	96.6	1744	95.1	0.024
Yes	156	4.1	67	3.4	89	4.9	
**Compliance to hygiene measure**							
0 (bad)	356	9.4	107	5.4	249	13.6	0.000
1	771	20.3	377	19.1	394	21.5	
2	1724	45.3	938	47.6	786	42.9	
3 (good)	952	25.0	548	27.8	404	22.0	
**Replacement of CL at intervals longer than recommended**							
No	2031	53.4	1084	55.0	947	51.7	0.038
Yes	1772	46.6	886	45.0	886	48.3	
**CL wear overnight**							
No	2272	59.7	1184	60.1	1088	59.4	0.640
Yes	1531	40.3	786	39.9	745	40.6	

CL, contact lens.

Crude and multivariate-adjusted ORs are shown in Table 3. Lens type was not associated with adverse events related to CL use. Factors associated with an increase in CL related eye complications include quarterly replacement of CL versus daily CL replacement, longer daily wear duration, CL purchase at physical shops and/or over the internet versus purchases at eye clinics/hospitals, poor compliance to recommended hygiene procedures, replacement of CLs at longer intervals than recommended, and sleeping in CLs.

**Table 3:** Factors associated with adverse events-related to CL use.

Variables	Crude ORs	95% CIs		*P* values	Adjusted ORs	95% CIs		*P* values
		Lower Limit	Upper Limit			Lower Limit	Upper Limit	
**Lens type**								
Transparent	1	−	−	−	1	−	−	−
Cosmetic	1.11	0.97	1.27	0.122	0.95	0.81	1.11	0.496
**Replacement schedule**								
Daily	1	−	−	−	1	−	−	−
Bi-weekly	1.01	0.86	1.17	0.930	0.88	0.73	1.05	0.152
Monthly	1.38	1.10	1.73	0.005	1.20	0.95	1.53	0.133
Quarterly	2.26	1.17	4.38	0.016	2.07	1.05	4.08	0.036
Annually	1.36	0.99	1.86	0.054	1.24	0.90	1.72	0.192
Others	1.52	0.77	2.98	0.228	1.61	0.80	3.21	0.180
Age (per year)	0.98	0.97	0.99	0.000	0.99	0.98	1.01	0.385
Total experience (per year)	0.99	0.98	0.998	0.018	1.00	0.98	1.01	0.556
Daily wear time (per hour)	1.05	1.03	1.07	0.000	1.03	1.01	1.05	0.001
**Location of purchase**								
Eye clinic/hospital	1	−	−	−	1	−	−	−
Physical shop	1.36	1.13	1.62	0.001	1.28	1.06	1.54	0.009
Internet	1.31	1.13	1.52	0.000	1.23	1.05	1.45	0.012
**Stacking of CLs**								
No	1	−	−	−	1	−	−	−
Yes	1.24	0.92	1.66	0.160	0.93	0.63	1.38	0.719
**CL exchange with friends**								
No	1	−	−	−	1	−	−	−
Yes	1.26	0.91	1.76	0.160	0.98	0.63	1.51	0.917
**Compliance to hygiene measure**								
0 (Bad)	1	−	−	−	1	−	−	−
1	0.73	0.57	0.95	0.017	0.88	0.67	1.15	0.334
2	0.54	0.43	0.68	0.000	0.75	0.58	0.98	0.033
3 (good)	0.56	0.43	0.71	0.000	0.71	0.54	0.94	0.017
**Replacement of CL at intervals longer than recommended**								
No	1	−	−	−	1	−	−	−
Yes	1.76	1.54	2.02	0.000	1.53	1.30	1.79	0.000
**CL wear overnight**								
No	1	−	−	−	1	−	−	−
Yes	1.55	1.36	1.78	0.000	1.25	1.07	1.45	0.004

CI, confidence interval; OR, odds ratio; CL, contact lens.

## Discussion

The research group of the Ministry of Health, Labor and Welfare, Japan, investigated the risk factors on CL-related eye problems among middle school and high school students throughout Japan. In that survey^13^, a higher school grade and female sex, as well as poor compliance with CL care, were identified as risk factors for CL-related eye problems. Therefore, we restricted the study to females.

Overall, 34.5% of CL wearers experienced eye problems, which was consistent with other studies^14–16^. Daily disposable CL wearers comprised 59% of our sample, but 82% were not considered compliant. In this study, poor compliance included people who reuse daily disposable CLs. This finding likely is not unique to our study. In the US, about 60% of daily disposable CL wearers store their CL in a case more than a single day. Among this group, over 80% extend their use of daily disposable CL by storing them in tap water^15^.

Our analysis also revealed that there was no significant difference in the frequency of CL-related eye disorders between CCL and transparent CL groups. This result was contrary to our expectations as we expected there would be a different set of CL-related habits between the two groups. In fact, as Tables 2 and 3 show, the CCL wearers seemed to live a less risk-averse lifestyle regarding CL habits. In the multivariate analysis model, factors associated with increased frequency of eye complications included quarterly replacement schedule versus daily disposable use, longer wear times, purchases of CLs from shops and over the internet rather than from health professionals, poor compliance to hygiene procedures, extending the recommended CL replacement schedule, and the wearing of CLs while sleeping. A majority of the findings above are consistent with previous publications.

The replacement schedule is an important factor used to predict eye complication risk. In theory, the risk of adverse events related to CL use for daily disposable CLs was less than any other type of CL. The decrease in risk associated with daily disposable CLs is attributed to the avoidance of CL solution interaction, CL case contamination, and reduced likelihood of the introduction of biofilm to the CL or case. Despite these assumed benefits of disposable daily CL, a significant difference was not observed between daily disposable CLs and other CLs. Our results showed similar safety or risk to other CLs when users behaved similarly. The reason for significantly higher OR for eye complication for quarterly CLs was unclear and should be investigated further. But considering a wide 95%CI of quarterly CL use amongst survey participants, a major reason might be due to the small number of them recruited in this study. Another confounding factor we did not consider was the potential that CL properties might be affected by other cosmetic item uses such as eyelash liners and/or hand creams that could result in eye damage.

Longer daily usage of CLs was associated with more eye complications in our analysis. The reason might be due to the potential that users with longer daily wear times included both extended wear CL users and overnight wearers. Wearing CLs for long periods can cause dry eye^17^, which has symptoms that include foreign body sensation, dryness, eye strain, and blurred vision^18^. Overnight CL wearing was also a well-known independent risk increase behavior^19,20^, and our result was consistent with those findings (OR 1.25 [95%CI 1.07–1.45]). In a biological sense, extended CL wear and overnight CL wear were associated with the presence of IL-8 and epidermal growth factor^21,22^, both of which indicate mechanical trauma.

CL purchases by internet order^19^ or at unlicensed vendor shops^23^ were reported to increase the risks of adverse events because these purchase locales never provide eye examinations and/or sufficient counseling. Our results are consistent with these findings, and we implore the need to improve health literacy for CL users. Regulation of purchase channels for CLs would be a considerable challenge for public health ophthalmology but may reduce the incidence of adverse events related to CL use.

The Centers for Disease Control and Prevention recommends that CL wearers should follow three hygiene-related behaviors: not to use tap water to store/rinse CLs, to use disinfecting solution properly, and to use cases properly^15^. In line with these findings, we obtained a convincing result of higher ORs for “poor compliance to hygiene procedure.” However, even greater ORs would likely be observed if we included more detailed hygiene-related behavior questions, such as handwashing habits when handling CLs, personal practice of rubbing CLs, and/or the storage case after removing CLs.

Stacking one contact on top of another could change the fitting of CL and would reduce how much oxygen the cornea receives throughout the duration of CL wear. In our study, however, stacking behavior was not associated with CL-related eye complications. This result is likely due to relatively short wear time from either poor vision and/or discomfort. Exchanging CLs among friends was not related to adverse events, either. The reason for the lack of eye complication as a result of CL sharing might be due to the exchange of a new pack of CLs with similar power and curve, or the friend wore the CLs for a short amount of time.

Age and total years of experience were not associated with a difference in the rate of complications. The reason might be related to the correlation between age and experience. Regardless of the potential correlation, the significant relationship was not observed (results not shown) even when either experience or age was selected in multivariate logistic regression analysis. This result did not confer a greater level of safety for experienced CL users and may be influenced by survival bias, meaning that only subjects who had no complications in the past with CLs kept using CLs.

A major strength in this study was the large sample size that was recruited nationwide in Japan. We do note that there were several limitations to this study. Selection bias could be a major concern for generalizability since wealthier, and more educated individuals might have more access to the online survey^24,25^. If our sample included poorer and less educated individuals, the proportion of adverse events would be more significant, especially as a result of CL usage. Second, CL-related eye complications ranged from slight to severe, sight-threatening conditions. For this study, we considered all eye complications to be either present or not. Due to the survey aspect of this study, it would not be realistic to receive the information about medical diagnosis and severity from non-medical professionals and may result in uncontrollable information bias. Third, we had no data on lens materials or CL brands for survey respondents. In our analysis, there was no significant difference in CL-related eye complications between transparent and CCLs. In general, those who participate in the health survey are said to have greater health literacy. These kinds of people might develop customer loyalty to brands that provide consistent, high-quality products. If counterfeit brands were on the market, the CCL-wearing group, especially young females, might suffer disproportionately from eye injuries due to exposed pigments and surface roughness^26,27^ or from microbial contamination^28^. Fourth, we included confounding factors determined from our literature review. Confounders, such as refraction, lens fitting, tobacco use, systemic disease, and socioeconomic status, were not included. Due to the nature of online surveys, there might be a higher chance that some questions would be ignored or left unanswered. To avoid this bias, we selected only the most important questions. Fifth, this analysis does not tell us which hygiene-related behaviors led to specific adverse events. We created a new variable by combining three hygiene-related practices because of the high correlation between three predictive variables. This relationship may lead to unreliable estimates of ORs.

Several studies indicated a higher risk of eye complications as a result of CCL use. Our study found that there was no significant difference in adverse events related to CL use between the transparent CL group and the CCL group. CL-related complications are preventable by the improvement of CL user behaviors. All CL users should reduce daily wear time by avoiding overnight wearing, and CL users should maintain compliance to recommended hygiene procedures and lens disposable/replacement schedules under the supervision of eye health professionals. The findings from this research support the mission of public health ophthalmology that aims to create awareness of adverse events related to CL and to improve CL user behaviors to maintain their health and safety. Eye professionals should prescribe CLs as they can examine the ocular surface, choose properly fitting CLs, and advise about regular eye examination and lens management. A stronger relationship between ophthalmologists and CL users could improve lens management and eye health.

## Data Availability

The data that support the findings of this study are available from the corresponding author, KO, upon reasonable request.
